# Comparative anatomy of the encephalon of new world primates with emphasis for the *Sapajus* sp

**DOI:** 10.1371/journal.pone.0256309

**Published:** 2021-09-01

**Authors:** Tainá de Abreu, Maria Clotilde Henriques Tavares, Rafael Bretas, Rosângela Correa Rodrigues, Alcides Pissinati, Tales Alexandre Aversi-Ferreira

**Affiliations:** 1 Morphology Museum, Federal university of Tocantins, Palmas, Tocantins, Brazil; 2 Graduate School of Animal Biology, Institute of Biology, University of Brasilia, Darcy Ribeiro Campus, Brasília, DF, Brazil; 3 Laboratory for Symbolic Cognitive Development, RIKEN Center for Biosystems Dynamics Research, Kobe, Japan; 4 Department of Biological Sciences, State University of Feira de Santana, Feira de Santana, Bahia, Brazil; 5 Centro de Primatologia do Rio de Janeiro (CPRJ/INEA), Estrada do Paraíso s/n. Guapimirim, Rio de Janeiro, Brazil; 6 Laboratory of Biomathematics and Physical Anthropology, Biomedical Sciences Institute, Federal University of Alfenas, Alfenas, MG, Brazil; University Ceuma, BRAZIL

## Abstract

Studies about the anatomy of the New World Primates are scarce, mainly comparative neuroanatomy, then a morphological comparative analysis about the tropical Primates were performed and a effort was made for an Old World Primates and modern humans relationship for the obtained data; plus, comments about behavior e and allometry were performed to try link the high cognition and abilities of the *Sapajus* with the neuroanatomical results, however, despite the deep neuroanatomic data obtained, we do not found an intrinsic relation to explain that.

## Introduction

Human evolutionary history is directly associated with brain development, considering that the construction of society depends on the high cognition observed in the humankind [1]. Indeed, the human brain is the most sophisticated instrument of cognition and, putatively, the most complex material structure known [2]. Therefore, the neural system has been studied extensively in multidisciplinary domains, but many questions about brain functions remain answered [3]

One of the approaches to understand the cerebral physiology is to study the historical/archaeological data from primitive humans and to observe and to use extant models of evolution, such as other primates, to understand the anatomical features that made humans a species with higher intelligence showing unique characteristics such as language, self-ornamentation, and artistic and religious manifestations [1], inter alia. However, the use of ancestral fossils for brain studies is limited because soft tissues are not well conserved. Therefore, only the bone impressions of the encephalon remain, which are insufficient for a deep understanding of its anatomical history. Therefore, chimpanzees and gorillas are the most studied models for human evolution, social cognition, hand abilities, behavioral flexibility, and tool use, certainly because they are the most cognitive non-human primates and closer to humans in terms of phylogenesis, brain size [1] and DNA similarities [4]; in terms of phylogenetic proximity [5] chimpanzees, bonobos, gorillas, and orangutans are considered, in this order, as the main models to study the human evolutionary history.

Various studies about evolution of the mind and intelligence of modern humans are based mainly on the behavior of apes and other primates—social and solitary species—using tests of cognition about memory and learning; however, for the neurophysiological research, macaques are the main studied primates [6, 7]. Despite that, data of gross anatomy of the cerebrum are scarce, especially when considering comparative studies.

Behavioral (ethological) studies are sometimes performed before a structural analysis [8] of the animals, leading to some misinterpretations [8, 9]. However, studies do not indicate a positive correlation of brain anatomy with behavior, cognition, or other complex abilities, except, perhaps, with brain size [1, 10, 11]; indeed, proportional analysis indicates no significant difference between the cerebral mass of modern humans and baboons, for instance [12].

Thus, a study of the gross anatomy of the brain performed in many primate species from different taxa may generate a negative answer for the following question: “Does the brain anatomical structure correspond the level of cognitive development in phylogenetic evolution?” Such a study is essential to better understand the relationship between cognitive development and brain anatomy; however, to date, such a study has not been performed in the species we describe here.

It is well established that some gyri and sulci are common for all primates, whereas others exist in non-human primates but not in modern humans [13]. However, an accurate comparative study of the brains of neotropical primates, Old World primates, apes, and modern humans, analyzing aspects such as the main gyri and sulci, brain size, and gyrencephaly has not yet been performed.

Among all primates, the neotropical ones are scarcely studied, except for the genus *Sapajus* (bearded capuchins), which, remarkably and unexpectedly, exhibit characteristics such as memory, cognition, social behavior, and tool use similar to chimpanzees [9, 13–28] a big brain in relation to their body mass [11, 20]; and high motor development [29, 30]. Additionally, there is some research on the brain of *Saimiri collinsi* [31], *Sapajus apella* (formerly *Cebus apella*) [11, 32] and *Callithrix* sp. [33], but the studies did not include a detailed comparative anatomy or investigate the correlations with behavior and evolution. Furthermore, a study investigated the brain anatomy of *Sapajus*, but some information was missing [11].

Therefore, the present study aims to perform a comparative analysis of experimental data of the main aspects of the gross anatomy of the brain of neotropical primates such as *Callithrix penicillata*, *Saimiri ustus*, *Brachyteles arachnoides*, and *Sapajus libidinosus* and data from previous studies on *Saimiri collinsi*, *Galago* sp., *Papio* sp., *Macaca* spp., *Pongo* sp., *Gorilla* spp., chimpanzees, and modern humans. Allometric measurements of the sulci along a straight line and along the sinuosities were performed to generate a dataset to facilitate comparison among the species in terms of the degree of gyrencephaly for dissected specimens; moreover, the encephalization quotient was calculated for neotropical primates to allow comparison with the data for other primates from previous studies. When possible, we investigated the association between the obtained data with the general behavioral aspects of the respective primate species.

## Results

### Macroscopic description of the encephalon of *Callithrix penicillata*

**Fig 1:**
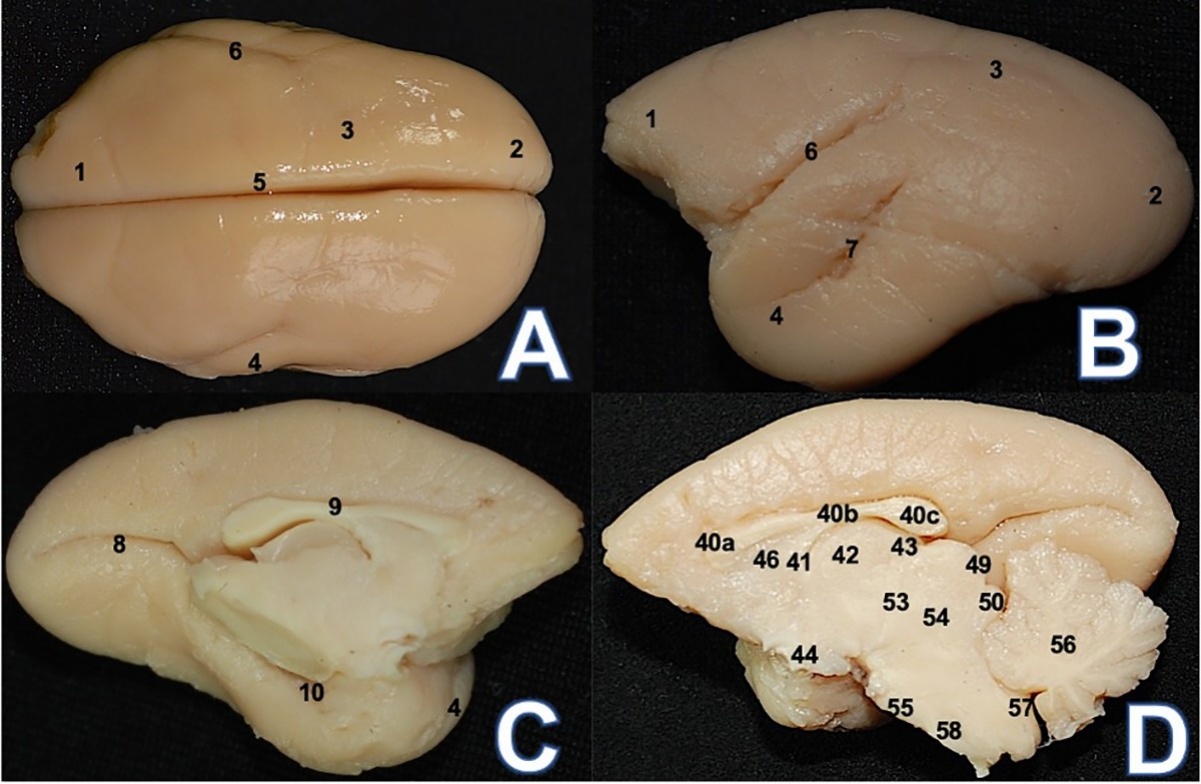
*Callithrix penicillata* (CP1) encephalon. (A) Superior view: 1. Frontal lobe 2. Occipital lobe 3. Parietal lobe 4. Temporal lobe 5. Sagittal/longitudinal fissure 6. Lateral sulcus (B) Lateral view: 7. Superior temporal sulcus (C) Medial view: 8. Calcarine sulcus 9. Sulcus of the corpus callosum 10. Hippocampal sulcus; (D) Medial view: 40a. corpus callosum genu 40b. corpus callosum trunk 40c. corpus callosum splenium 41. Fornix 42. Thalamus 43. Epiphysis 44. Hypothalamus 46. Septum pellucidum 49. Superior colliculus 50. Inferior colliculus 53. Metathalamus 54. Mesencephalon 55. Pons 56. Cerebellum 57. Ventriculus quartus 58. Bulb.

The height, length, and width measurements of *C*. *penicillata* brain were 18.08, 29.10, and 21.43 mm, respectively; its volume was 0.6 mL, and EQ was 1.7. The lissencephaly of the frontal, parietal and occipital lobes made the delimitation of cerebral hemispheres challenging; however, it was possible to identify the longitudinal fissure and lateral and temporal sulci on the convex aspect of the brain (Fig 1A). The lateral sulcus is a small sulcus in the oblique direction. The superior temporal sulcus in the temporal lobe is visible only in the lateral view, in a direction approximately parallel to lateral sulcus (Fig 1B).

On the medial aspect, it was possible to visualize the calcarine, rhinal, hippocampal sulci and sulcus of the corpus callosum. This region presents few gyri and could be considered as lissencephalic (Fig 1C). The posterior portion of the sulcus of the corpus callosum ends at the junction between the hippocampal and calcarine sulci (Fig 1C). The rhinal sulcus is a short, shallow and arcuated sulcus that is located posteriorly to the temporal lobe; the hippocampal sulcus—found posteriorly to the calcarine sulcus and sulcus of the corpus callosum—terminates in the uncus (Fig 1C).

The telencephalic sulci shows negligible gyrencephaly or degree of sinuosity (Table 1), indicating a straight trajectory, and therefore exhibits few gyri. For instance, neither the central sulcus nor the precentral and postcentral sulci were evident, and thus the precentral and postcentral gyri could not be observed (Fig 1A and 1B).

**Table 1:** Presence of the main sulci in the medial region of the brain of modern humans and other primates, in special those belonging to the genera *Pan*, *Papio*, *Macaca*, *Galago*, *Ateles*, *Cebus*, and *Sapajus*.

Sulcus	*Galago*	*Callithrix*	*Saimiri*	*Sapajus*	*Alouatta*	*Ateles*	*Brachyteles*	*Macaca*	*Papio*	*Pan*	*Homo*
Longitudinal	X	X	X	X	X	X	X	X	X	X	X
Central	-	-	X	X	X	X	X	X	X	X	X
Precentral	-	-	-	X	X	X	X	X	X	X	X
Postcentral	-	-	-	X	X	X	X	X	X	X	X
Inferior frontal	-	-	-	X	X	X	X	X	X	X	X
Lateral	X	X	X	X	X	X	X	X	X	X	X
Superior temporal	-	X	X	X	X	X	X	X	X	X	X
Inferior temporal	-	-	-	X	X	X	X	X	X	X	X
Lunatus	-	-	X	X	X	X	X	X	X	X	X
Inferior occipital	-	-	-	X	X	X	X	X	X	X	X
Cingulate	X	-	X	X	X	X	X	X	X	X	X
Sulcus of the corpus callosum	Not found	X	X	X	Not found	Not found	X	X	Not found	Not found	X
Rostral	-	-	-	X	X	X	X	X	X	X	X
Subparietal	-	-	X	X	-	-	-	X	X	X	X
Parieto-occipital	-	-	X	X	X	X	X	X	X	X	X
Calcarine	X	X	X	X	X	X	X	X	X	X	X
Calcarine ramus	-	-	X	X	X	X	X	X	X	X	*
Occipitotemporal	-	-	-	X	X	X	-	X	X	X	X
Hippocampus	X	X	X	X	X	X	X	X	X	X	X
Collateral	-	-	X	X	X	X	X	X	X	X	X
Rhinal	X	X	X	X	X	X	X	X	X	X	X

* Variations found in Tamraz and Comair [34].

- data not found in the literature.

The other encephalic structures of *C*. *penicillata* are similar to the corresponding structures in the human brain but with different anatomical organization; for instance, the corpus callosum is appears straighter than other primates, as indicated by the sinuosity of the sulcus of the corpus callosum (Table 1), and the angle of the encephalic trunk in relation to thalamus is greater than that in humans.

No specific differences were observed in other inferior structures, such as the pellucid septum; corpus callosum that presents genu, trunk, splenium, and an almost imperceptible rostrum; diencephalon; mesencephalon; pons; cerebellum; and medulla oblongata (Fig 1D).

Tables 1 and 3–5 show the absolute measures of the sulci, gyri, encephalic trunk, and other structures.

### Macroscopic description of the encephalon of *Saimiri ustus*

The height, length, and width measurements of the *Samiri ustus* encephalon were 28.48, 44.38, and 34.10 mm, respectively. The encephalon volume was 22 mL, and EQ was 2.25.

**Fig 2:**
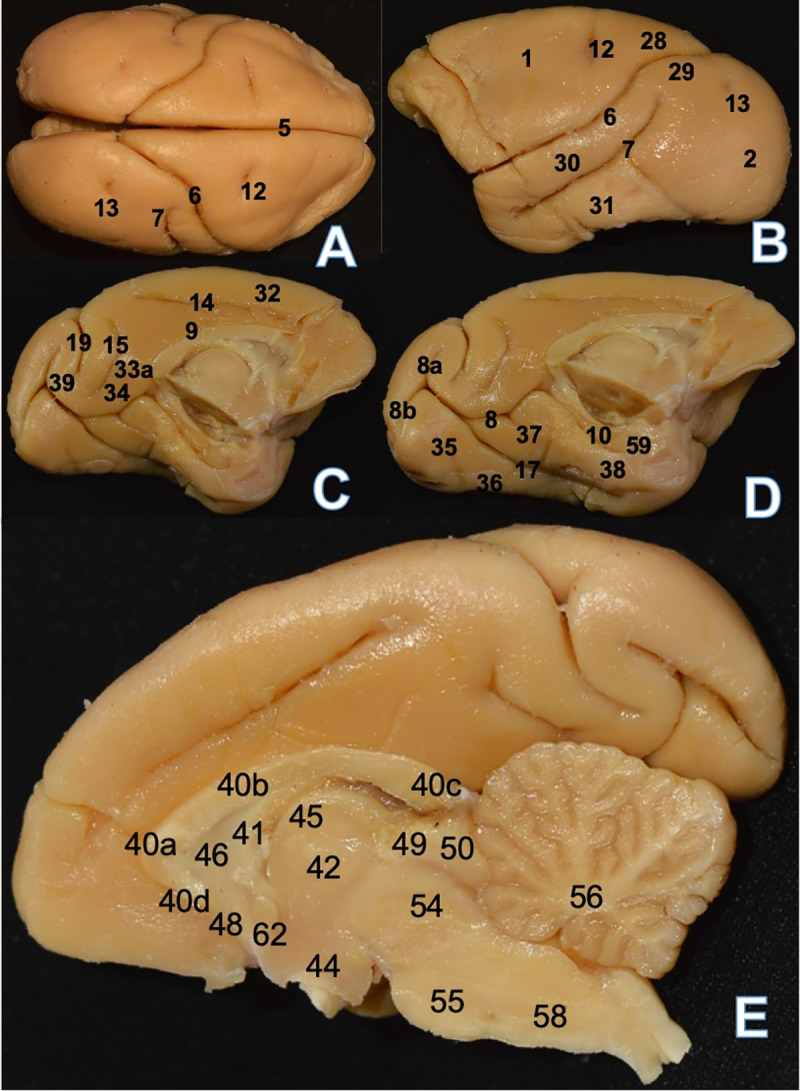
*Saimiri ustus* (SU1) encephalon. (A) Superior view: 5. Sagittal/longitudinal fissure 6. Lateral sulcus 7. Superior temporal sulcus 12. Central sulcus 13. Lunatus sulcus. (B) Lateral view: 1. Frontal lobe 2. Occipital lobe 6. Lateral sulcus 7. Superior temporal sulcus 12. Central sulcus 13. Lunatus sulcus 28. Parietal rostral gyrus 29. Parietal caudal gyrus 30. Superior temporal gyrus 31. Inferior temporal gyrus. (C) Medial view: 9. Sulcus of the corpus callosum 14. Cingulate sulcus 15. Subparietal sulcus 19. Parieto-occipital sulcus 32. Medial frontal gyrus 33a. Cingulate gyrus isthmus 34. Precuneus 39. Cuneus. (D) Medial view: 8. Calcarine sulcus 8a. Superior ramus 8b. Inferior ramus 10. Hippocampal sulcus 17. Collateral sulcus 35. Occipital gyrus 36. Fusiform gyrus 37. Lingual gyrus 38. Para hippocampal gyrus 59. Uncus. E. Medial view: 40a. Corpus callosum genu 40b. Corpus callosum trunk 40c. Corpus callosum splenium 40d. Corpus callosum rostrum 41. Fornix 42. Thalamus 44. Hypothalamus 45. Medullary stria of thalamus 46. Septum pellucidum 48. Anterior commissure 49. Superior colliculus 50. Inferior colliculus 54. Mesencephalon 55. Pons 56. Cerebellum 58. Bulb 62. Mammillary body.

There is a deep longitudinal fissure separating the hemispheres and a few sulci and gyri on the convex aspect (Fig 2A and 2B). The central sulcus is shallow and short, and anteriorly from it, the frontal lobe is lissencephalic. In contrast, the posterior parietal area presents two big gyri or lobules generated by the continuation of the lateral sulcus (Fig 2B)—the rostral and caudal lobules. The lateral sulcus is deep and separates the frontal, parietal, and temporal lobes; it is present in an inclined/oblique direction and continues in the medial aspect (Fig 2B).

The superior temporal sulcus in the temporal lobe is parallel to the extension of the lateral sulcus and generates two gyri: the superior and inferior temporal gyri (Fig 2B). The occipital lobe is lissencephalic on the convex aspect, but it is possible to observe a small depression called lunatus sulcus (Fig 2A and 2B). On the medial aspect, the sulcus of the corpus callosum and parieto-occipital, rhinal, hippocampal, collateral, and calcarine sulci are visible (Fig 2C and 2D).

The cingulate sulcus does not follow the arcuate curve of the corpus callosum; it is a straight sulcus in the frontal-to-parietal direction that delimits superiorly the cingulate gyrus, which is posteriorly separated from the occipital lobe by the subparietal sulcus (Fig 2C). The cingulate sulcus is delimited anterior-inferiorly by the sulcus of the corpus callosum and posterior-inferiorly by the calcarine sulcus (Fig 2D) in the region of the isthmus of the cingulate gyrus.

Posteriorly, the sulcus of the corpus callosum meets the calcarine sulcus. The corpus callosum presents a rostrum, genu, trunk, and splenium and the structure is somewhat arcuated (Fig 2D). Continuous with the lateral sulcus but medially, the parieto-occipital sulcus separates the parietal and occipital lobes; anterior to the parieto-occipital sulcus is the subparietal sulcus, which is delimited anteriorly by the precuneus and posteriorly by the isthmus of the cingulate gyrus (Fig 2C).

The calcarine sulcus begins in the temporal lobe on the medial aspect and continues to the occipital lobe, where it bifurcates into superior and inferior rami; it joins the sulcus of the corpus callosum anteriorly (Fig 2D). Situated between the parieto-occipital sulcus and superior ramus of the calcarine sulcus is the cuneus gyrus (Fig 2C). The collateral sulcus begins from the calcarine sulcus in the occipital lobe and ends in the anterior medial region of the temporal lobe. It delimits the lingual gyrus in its posterior inferior portion and the parahippocampal gyrus in its anterior inferior portion (Fig 2D).

The lingual gyrus is found between the calcarine and collateral sulci; it is delimited superiorly by the hippocampal sulcus. Located below the collateral sulcus is the fusiform gyrus (Fig 2D). The hippocampal sulcus begins posteriorly in the bifurcation between the sulcus of the corpus callosum and the anterior portion of the calcarine sulcus and terminates cranially below the uncus (Fig 2D).

The diencephalon presents structures and features similar to those observed in humans and other primates; the encephalic trunk is present at an angle greater than 90° in relation to a vertical axis passing through the telencephalon, and it exhibits the general characteristics found in humans and other non-human primates (Fig 2E). Evidently, the absolute measurements of the brain structures differ among these species.

Tables 3–5 present the absolute measurements of the sulci, gyri, encephalic trunk, and other structures.

### Macroscopic description of the encephalon of *Sapajus libidinosus*

**Fig 3:**
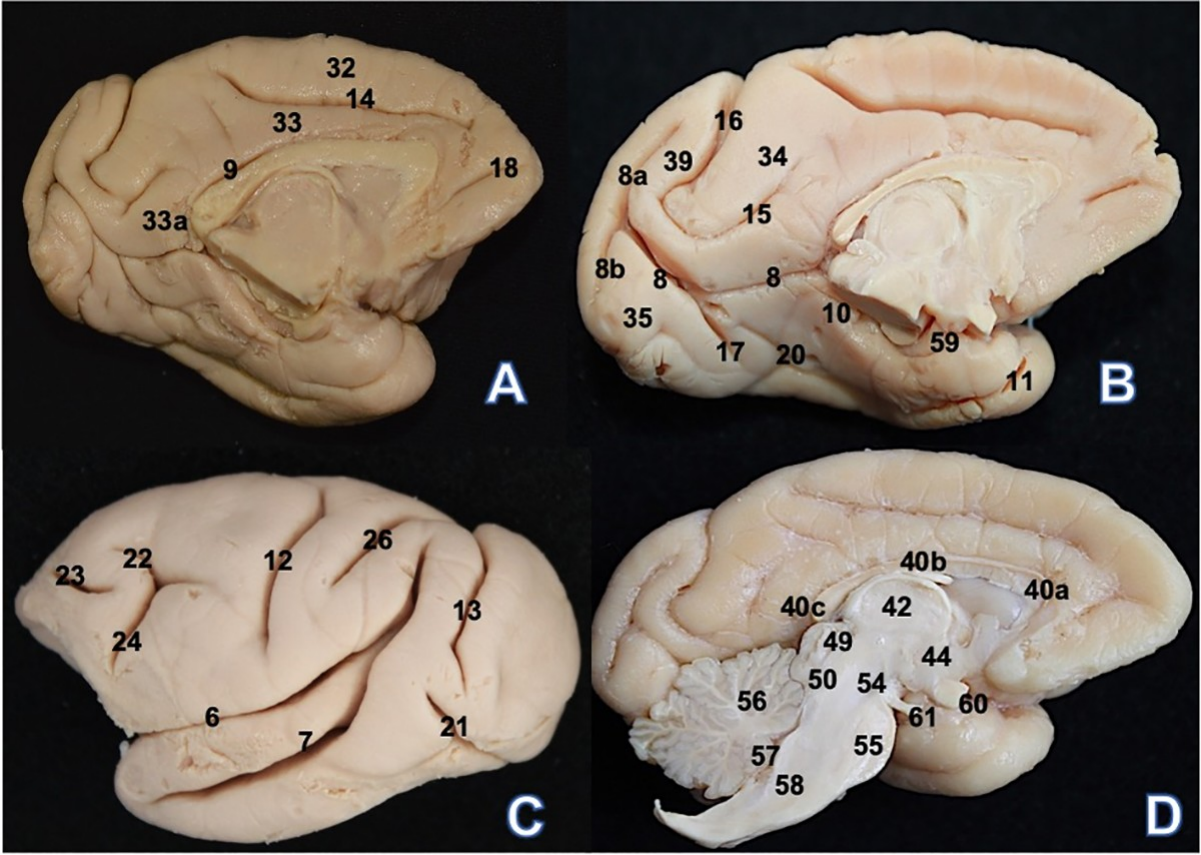
*Sapajus libidinosus* (SP1) encephalon. (A) Medial view: 9. Sulcus of the corpus callosum 14. Cingulate sulcus 18. Rostral sulcus 32. Medial frontal gyrus 33. Cingulate gyrus 33a. Cingulate gyrus isthmus. (B) Medial view: 8. Calcarine sulcus 8a. Superior ramus 8b. Inferior ramus 10. Hippocampal sulcus 11. Rhinal sulcus 15. Subparietal sulcus 16. Parieto-occipital sulcus 17. Collateral sulcus 20. Occipital-temporal sulcus 35. Occipital gyrus 59. Uncus 39. Cuneus. (C) Lateral view: 6. Lateral sulcus 7. Superior temporal sulcus 12. Central sulcus 13. Lunatus sulcus 21. Inferior occipital sulcus 22. Longitudinal superior sulcus 23. Longitudinal inferior sulcus 24. Vertical sulcus 26. Postcentral sulcus. (D) Medial view: 40a. Corpus callosum genu 40b. Corpus callosum trunk 40c. Corpus callosum splenium 42. Thalamus 44. Hypothalamus 49. Superior colliculus 50. Inferior colliculus 54. Mesencephalon 55. Pons 56. Cerebellum 57. Ventriculus quartus 58. Bulb 60. Optic chiasm 61. Oculomotor nerve.

In this study we focus our description on the macroscopic features of the lateral part of the *Sapajus libidinosus* brain, diencephalon and encephalic trunk, with limited comments on the medial part, already comprehensively described be Pereira-de-Paula [11]. On the medial aspect of *Sapajus libidinosus* encephalon, superiorly, the cingulate sulcus and gyrus are visible (Fig 3A). The cingulate gyrus is delimited inferiorly by the sulcus of the corpus callosum and superiorly by the cingulate sulcus. Located above the cingulate sulcus is the medial frontal gyrus (Fig 3A).

The cingulate sulcus is present anterior and superior to the corpus callosum; it courses in the anterior-to-posterior direction at a small inclination angle. Approximately in the posterior third section, it sharply ascends to the superior border and terminates before reaching the superior border; the sharply ascending section is called marginal ramus (Fig 3A).

A small and solitary sulcus is observed in the medial and anterior part of the frontal lobe, here designated as the rostral sulcus (Fig 3A). The sulcus of the corpus callosum contours the corpus callosum; it starts above the rostrum and terminates below the splenium, where it joins the calcarine and hippocampal sulci (Fig 3B). The calcarine sulcus, another sulcus that starts close to the splenium, courses in the posterior direction towards the occipital lobe, where it bifurcates into superior and inferior rami (Fig 3B). Lateral and inferior to the anterior part of the calcarine sulcus is the lingual sulcus, which also delimits the isthmus of the cingulate gyrus. The middle and superior parts of the calcarine sulcus delimit the cuneus in the superior part of the occipital lobe (Fig 3B). The cuneus is anteriorly delimited by the occipital sulcus (Fig 3B).

At the beginning of the calcarine sulcus running in the anterior direction in the medial superior part of the temporal lobe is situated the hippocampal sulcus, which terminates in the uncus (Fig 3B). The collateral sulcus begins inferior to the middle third of the calcarine sulcus and delimits the anterior section of the lingual gyrus and the posterior section of the fusiform gyrus. The occipital-temporal sulcus is located anterior and inferior to the collateral sulcus and terminates approximately inferior to the starting point of the rhinal sulcus. The rhinal sulcus is shallow and situated in the middle of the temporal pole (Fig 3B).

The fusiform gyrus is delimited inferiorly by the occipital inferior sulcus, which contours the inferior portion of the occipital lobe and appears on the convex aspect of this lobe, terminating in the posterior part of the temporal lobe (Fig 3C). Located inferiorly in the temporal lobe is the occipital-temporal sulcus, which delimits the parahippocampal gyrus inferiorly and separates this gyrus from the inferior temporal gyrus (Fig 3B). The parieto-occipital sulcus separates the parietal and occipital lobes medially and is continuous with the subparietal sulcus in most of the antimeres [12, 14] (Fig 3B).

The structures present in the diencephalon and encephalic trunk are very similar to the corresponding structures found in other non-human primates and humans, but the absolute dimensions and angulation in relation to a vertical axis through the telencephalon are different (Fig 3D).

Tables 2–6 present the absolute measurements of the sulci, gyri, encephalic trunk, and other structures.

**Table 2:** Relative measurements of the sulci, gyri, encephalic trunk, and other structures as a function of brain length (length of the cerebral hemisphere from the frontal to the occipital pole for each species–RM (cm), or relative measure).

Structure measurements	*Callithrix penicillata*	* *RM	*Saimiri ustus*	RM	*Sapajus libidinosus*	RM	*Brachyteles arachnoides*	* *RM	*sd*
Length of cerebral hemisphere from frontal to occipital pole	2.91		4.43		5.99		7.95		
Cerebral hemisphere height (Upper-Lower)	1.80	0.62	2.84	0.64	3.61	0.60	4.77	0.6	0.01
Brain width (lateral-lateral)	2.14	1.19	3.41	1.20	4.57	1.27	5.51	1.15	0.04
Olfactory bulb length	0.69	0.32	1.16	0.34	1.49	0.33	0.00	0	0.16
corpus callosum length	1.24	1.80	1.94	1.67	2.59	1.74	3.07	0.38	0.67
Distance from anterior end of corpus callosum to frontal pole	0.55	0.44	0.85	0.44	1.15	0.44	1.61	0.52	0.04
Distance from posterior end of corpus callosum to occipital pole	1.17	2.13	1.86	2.19	2.21	1.92	3.09	1.91	0.13
Anterior commissure (longitudinal diameter)	0.17	0.15	0.30	0.16	0.25	0.11	0.20	0.06	0.04
Length of thalamus (anteroposterior) in medial view	0.61	3.59	0.79	2.63	1.00	4.00	1.23	6.15	1.48
Thalamus height in medial view	0.28	0.46	0.61	0.77	0.73	0.73	0.92	0.74	0.14
Midbrain height (upper-lower)	0.54	1.93	0.51	0.84	0.57	0.78	1.10	1.19	0.52
Pons length (Anteroposterior)	0.57	1.06	0.76	1.49	0.86	1.51	1.31	1.190	0.22
Pons width (laterolateral)	0.89	1.56	1.15	1.51	1.60	1.86	2.27	1.73	0.15
Pons height	0.40	0.45	0.54	0.47	0.98	0.61	1.37	0.603	0.08
Bulb length (Anteroposterior)	0.40	1.00	0.71	1.31	0.68	0.69	1.15	0.83	0.26
Cerebellum width (lateral-lateral)	1.41	3.53	2.26	3.18	3.41	5.01	4.87	4.23	0.81
Cerebellum length (upper-lower)	0.95	0.67	1.42	0.63	1.35	0.40	2.95	0.60	0.12
Cerebellum height	1.09	1.15	1.48	1.04	1.77	1.31	2.10	0.71	0.25

**Table 3:** Absolute measurements (cm), of the distance between the main sulci and the occipital and frontal poles.

Sulcus	*Callithrix penicillata*		*Saimiri ustus*		*Sapajus libidinosus*		*Brachyteles arachnoides*	
	Distance to frontal pole	Distance to occipital pole	Distance to frontal pole	Distance to occipital pole	Distance to frontal pole	Distance to occipital pole	Distance to frontal pole	Distance to occipital pole
	Mean	Mean	Mean	Mean	Mean	Mean	Mean	Mean
Superior edge of central sulcus	-	-	2.23	2.19	3.73	3.36	3.56	4.33
Inferior edge of central sulcus	-	-	2.23	2.19	4.24	2.91	2.73	5.13
Central sulcus	-	-	2.55(±0.0)	1.88 (±1.64)	-	-	3.94(±2.55)	3.61(±2.19)
Postcentral sulcus	-	-	-	-	3.30 (±9.89)	1.56(±2.74)	3.82 (±0.13)	3.00(±2.14)
Inferior frontal sulcus	-	-	-	-	-	-	0.51	5.33
Lateral sulcus	0.54 (±0.73)	1.13 (±1.76)	0.68(±0.41)	1.01 (±2.06)	-	-	2.15 (±1.01)	1.09(±4.05)
Superior temporal sulcus	0.99 (±1.53)	1.26 (±1.46)	1.04(±1.46)	1.58 (±2.94)	1.16(±1.31)	1.61 (±3.35)	3.13 (±0.57)	2.31(±1.84)
Inferior temporal sulcus	-	-	-	-	1.15 (±1.16)	3.22 (±3.4)	3.03	3.98
Lunatus sulcus	-	-	3.78(±0.19)	0.43 (±0.22)	4.62 (±4.1)	1.61 (±3.59)	6.03 (±1.05)	1.33(±7.41)
Occipital inferior sulcus	-	-	-	-	4.14 (±4.61)	0.52 (±1.2)	-	-
Cingulate sulcus	-	-	1.02(±2.75)	1.88 (±0.63)	0.89 (±1.53)	2.129 (±3.63)	1.06 (±2.73)	2.62(±1.97)
Callosal sulcus	0.55 (±0.42)	1.09 (±0.61)	0.85(±0.43)	1.86 (±0.93)	1.156 (±1.12	2.19 (±0.93)	1.63(±1.03)	3.05(±0.28)
Rostral sulcus	-	-	-	-	0.38 (±1.48)	5.09 (±3.31)	2.3	6.60
Subparietal sulcus	-	-	3.02(±0.54)	1.04 (±0.84)	-	-	-	-
Parietooccipital sulcus	-	-	3.59(±0.82)	0.79 (±0.98)	4.02 (±3.65)	0.87 (±0.59)	5.80 (±2.39	2.09 (±0.72)
Calcarine sulcus	1.97 (±0.17)	0.19(± 0.83)	2.73(±1.21)	0.44 (±0.53)	3.91 (±2.63)	0.47 (±0.79)	4.92 (±0.95)	0.33 (±1.4)
Occipitotemporal sulcus	-	-	-	-	2.68 (±3.77)	1.43 (±1.79)	-	-
Hippocampal sulcus	1.13 (±1.97)	0.91(±0.52)	1.55(±1.66)	1.81 (±0.1)	2.45 (±2.86)	2.15 (±0.92)	3.37	3.01
Collateral sulcus	-	-	1.49 (±0.88)	0.88 (±0.59)	4.17 (±3.99)	1.06 (±1.37)	5.02 (±0.79)	1.45 (±2.19)
Rhinal sulcus	0.53 (±1.9)	1.79 (±1.49)	1.48 (±0.12)	2.65 (±2.36)	1.46 (±2.82)	3.07 (±3.2)	2.26 (±1.47)	3.98 (±0.94)

**Table 4:** Straight and sinuous measurements of the main sulci in *Callithrix penicillate*.

Sulcus	Straight measurement (cm) [1]				Sinuous measurement (cm) [2]				[1]/[2]
	RH		LH		RH		LH		
	Mean	Standard deviation	Mean	Standard deviation	Mean	Standard deviation	Mean	Standard deviation	
Lateral sulcus	1.06	0.13	1.09	0.94	1.16	0.53	1.24	2.89	0.89
Temporal sulcus	0.68	1.35	0.561	0.55	0.70	1.32	0.68	0.00	0.89
Sulcus of the corpus callosum	1.39	0.22	1.34	0.23	1.50	7.01	1.97	0.94	0.76
Calcarine sulcus	0.83	0.27	0.78	0.14	0.91	0.14	0.90	0.01	0.88
Hippocampal sulcus	1.10	1.03	0.81	1.37	1.19	1.87	0.93	1.73	0.89

RH: right hemisphere, LH: left hemisphere.

**Table 5:** Straight and sinuous measurements of the sulci on the medial aspect of the brain in *Sapajus libidinosus*.

Sulcus	Straight measurements (cm) [1]				Sinuous measurements (cm) [2]				[1]/[2]
	RH		LH		RH		LH		
	Mean	Standard deviation	Mean	Standard deviation	Mean	Standard deviation	Mean	Standard deviation	
Cingulate sulcus	3.41	2.52	3.2	2.75	3.52	2.61	3.41	3.49	0.96
Rostral sulcus	0.76	1.93	0.80	1.47	0.87	1.78	0.83	1.63	0.90
Sulcus of the corpus callosum	2.65	4.30	2.81	2.76	3.95	5.31	4.09	2.86	0.67
Calcarine sulcus	1.84	1.30	1.80	1.32	2.19	1.40	2.23	1.81	0.82
Hippocampal sulcus	1.53	2.77	1.50	1.60	1.83	3.09	1.87	1.31	0.82
Occipitotemporal sulcus	1.89	4.07	1.83	5.82	2.16	3.39	2.06	6.71	0.88
Rhinal sulcus	1.26	0.72	1.22	0.62	2.06	3.12	1.76	1.98	0.64
Collateral sulcus	0.89	2.05	0.96	3.20	1.06	2.92	1.15	3.53	0.83
Parieto-occipital + subparietal sulcus	1.41	0.80	1.35	0.86	3.02	4.18	2.82	4.89	0.47

RH: right hemisphere, LH: left hemisphere.

**Table 6:** Degree of sinuosity of the main sulci of primate species used in the present study.

Sulcus	*Callithrix penicillata*	*Saimiri ustus*	*Sapajus libidinosus*	*Brachyteles arachnoides*
Central sulcus	-	1	0.97*	0.84
Superior precentral sulcus	-	-	-	0.98
Inferior precentral sulcus	-	-	0.92**	0.92
Frontal sulcus	-	-	0.86***	0.91
Postcentral sulcus	-	-	0.89	0.97
Lateral sulcus	0.89	0.77	0.76*	0.76
Superior temporal sulcus	0.89	0.92	0.86	0.9
Inferior temporal sulcus	-	-	0.95	1
Lunatus sulcus	-	-	0.91	0.75
Inferior occipital sulcus	-	-	0.84	0.84
Rostral sulcus	-	-	0.9	1
Cingulate sulcus	-	0.98	0.96	0.86
Parieto-occipital sulcus	-	0.85	0.87*	0.81
Sulcus of the corpus callosum	0.76	0.56	0.67	0.6
Calcarine sulcus	0.88	0.8	0.82	0.94
Hippocampal sulcus	0.89	0.77	0.82	0.92
Rhinal sulcus	0.72	0.79	0.64	0.81

- Absent.

*Data from Pereira-de-Paula [11].

**Equivalent to vertical and longitudinal superior sulci described by Pereira-de-Paula [11].

*** Equivalent to longitudinal inferior sulci described by Pereira-de-Paula [11].

### Macroscopic description of the encephalon of *Brachyteles arachnoides*

**Fig 4:**
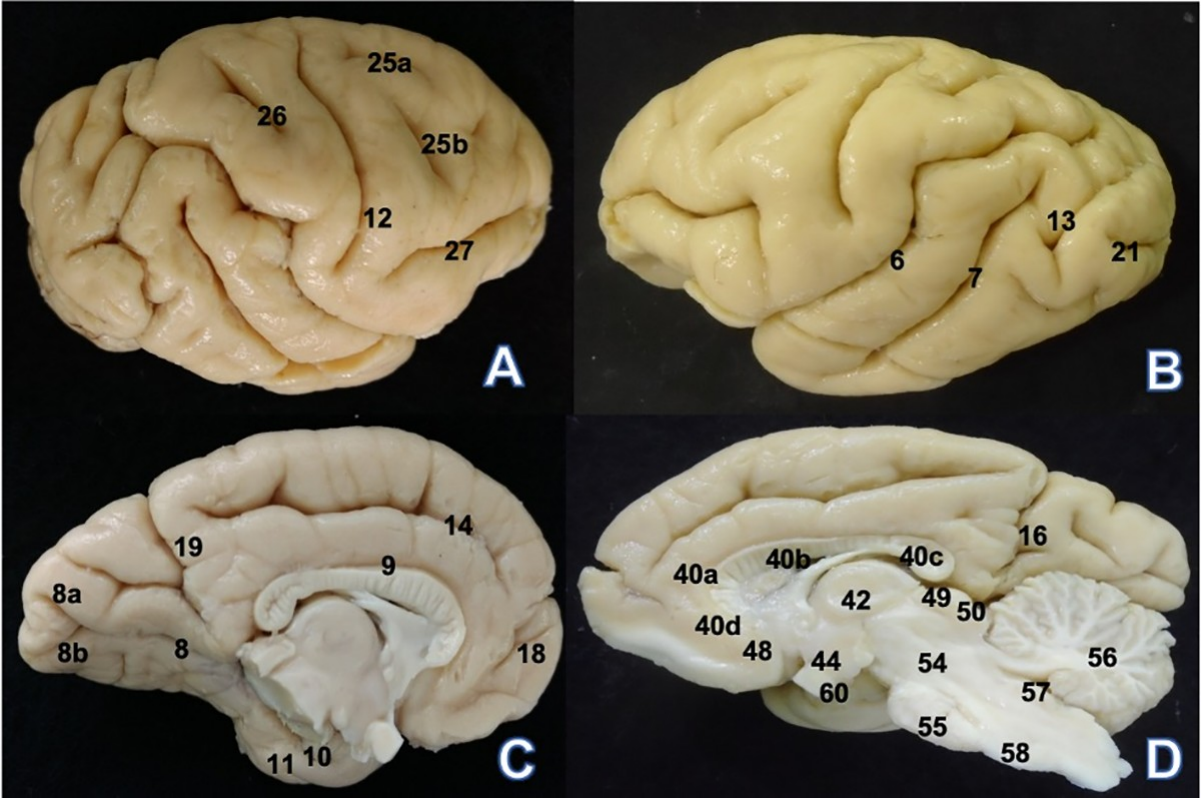
*Brachyteles arachnoides* (BA1) encephalon. (A) Lateral view: 12. Central sulcus 25a. Superior precentral sulcus 25b. Inferior precentral sulcus 26. Postcentral sulcus 27. Inferior frontal sulcus. (B) Lateral view: 6. Lateral sulcus 7. Superior temporal sulcus 13. Lunatus sulcus 21. Inferior occipital sulcus. (C) Medial view: 8. Calcarine sulcus 8a. Superior ramus 8b. Inferior ramus 9. Sulcus of the corpus callosum 10. Hippocampal sulcus 11. Rhinal sulcus 14. Cingulate sulcus 18. Rostral sulcus 19. Superior part of cingulate gyrus. (D) Medial view: 16. Parieto-occipital sulcus 40a. Corpus callosum genu 40b. Corpus callosum trunk 40c. Corpus callosum splenium 40d. Corpus callosum rostrum 42. Thalamus 44. Hypothalamus 48. Anterior commissure 49. Superior colliculus 50. Inferior colliculus 54. Mesencephalon 55. Pons 56. Cerebellum 57. Ventriculus quartus 58. Bulb.

Located centrally on the convex aspect of the *B*. *arachnoides* brain is the central sulcus, which separates the frontal and parietal lobes. It begins close to superior border of the hemisphere and terminates before encountering the lateral sulcus (Fig 4A). Three more sulci are observed on the convex aspect of the frontal lobe: the frontal, superior frontal, and precentral sulci (Fig 4A). The superior frontal sulcus has a longitudinal superior position close to the superior border of the hemisphere; oblique and almost vertical. The precentral sulcus is located superiorly to the inferior frontal sulcus (Fig 4A).

Located in the posterior part of the hemisphere behind the central sulcus is the postcentral sulcus in the parietal lobe, which is a small and deep sulcus partially parallel to the central sulcus (Fig 4A). A long, deep, and oblique sulcus marks the convex aspect of the hemispheres in the *B*. *arachnoides* brain—the lateral sulcus that courses across from the inferior to the superior border of the brain hemispheres and, after two curvatures, terminates in a bifurcation into the posterior region of the parietal lobe (Fig 4B).

Two sulci are observed in an inclined temporal lobe—the superior and inferior temporal sulci. The superior temporal sulcus is deep and runs parallel to the lateral sulcus across the temporal lobe, thus delimiting the superior and middle temporal gyri. A small, straight, and shallow inferior temporal sulcus scarcely separates the middle from the inferior temporal lobe (Fig 4B).

On the convex aspect, the lunatus sulcus, which separates the parietal and occipital lobes, presents differences between the right and left hemispheres; for instance, the lunatus sulcus joins the lateral sulcus in the right hemisphere not in the left hemisphere (Fig 4A and 4B). On the convex aspect, an inferior occipital sulcus appears in the middle part of the inferior occipital pole in both hemispheres; however, a superior occipital sulcus is prominent in the left occipital lobe but shallow and insignificant in the right occipital lobe (Fig 4B).

A straight sulcus observed on the medial aspect of the frontal lobe is the rostral sulcus (Fig 4C). The cingulate sulcus contours the cingulate gyrus from its anterior portion; differing in its posterior portion between the two hemispheres (Fig 4C). The parieto-occipital sulcus clearly separates the occipital and parietal lobes on the medial aspect (Fig 4C and 4D).

Contouring the corpus callosum is the arcuated sulcus of the corpus callosum; it ends at the junction between the hippocampal and calcarine sulci (Fig 4C). The hippocampal sulcus is arcuated in the cranial direction and ends in the uncus medially and inferiorly in the temporal pole (Fig 4C). From the occipital lobe, the calcarine sulcus emerges in a horizontal trajectory and terminates generating the superior and inferior rami prominently visible in the left hemisphere (Fig 4C).

Visible on the inferior surface of the occipital and temporal lobes is the collateral sulcus, including the inferior part of the hippocampal gyrus. The rhinal sulcus starts after the collateral sulcus in the anterior inferior part of the temporal lobe (Fig 4C).

Fig 4D shows structures such as the corpus callosum, diencephalon, and encephalic trunk. All these structures are anatomically similar to the corresponding structures in other primates; however, the angle of the encephalic trunk to a vertical axis through the telencephalon is greater than 90°.

Tables 3–6 show the absolute measurements of the sulci, gyri, encephalic trunk, and other structures.

### Allometric data of the studied encephalons

Data regarding the presence or absence of the main sulci in the medial region of the brain in the primate species investigated in the present study and previously published data were concatenated (Table 1).

The absolute measurements of the brain structures of the primate species studied in the present study are shown in part of the Table 2 and Tables 3–5. These measurements indicate the difference in the size of different structures, distance of sulci and gyri to the poles, and dimensions of the encephalon. These measurements indicate provide an approximatione of view about the size [(volume)] of some lobes.

A table and graph with straight versus curve dimensions of main gyrus were made to indicate the sinuosity degree among the primates studied this work (Table 6). For some primates was not possible to obtain all data.

Table 7 presents the relative values to facilitate a better comparison among the encephalon structures. These data permit to verify approximately the real relation of dimensions of the cerebral structures.

**Table 7:** Degree of sinuosity and degree of inclination of the central sulcus shown as the calculated Encephalization Quotient (EQ) for *Callithrix penicillata*, *Saimiri ustus*, *Sapajus libidinosus*, and *Brachyteles arachnoides* compared with the data for humans according Testut and Latarjet [13].

	*Callithrix penicillate*	*Saimiri ustus*	*Sapajus libidinosus*	*Brachyteles arachnoides*	*Homo sapiens*
Degree of sinuosity of central sulcus		1	0.97 [11]	0.84	0.76 [12]
Degree of inclination of central sulcus	-	0	-0.45	0.8	4.00 [12]
EQ	1.7	2.25	2.25–2.40	1.74	7.4–7.8

The EQ values were calculated for the primate species included in the present study and for data obtained from previous studies (Table 8).

**Table 8:** Encephalon mass, height, length, width, and Encephalization Quotient (EQ) of primate species studied in the present study and previous studies.

Subject	Mass (g)	EQ	Height (cm)	Length (cm)	Width (cm)
*Galago*	4.7	-	0.15	0.27	2.16
*Callithrix*	7	1.7*	1.78	2.95	2.14
*Saimiri*	24.12	2.25	2.84	4.43	3.41
*Sapajus*	71.06	2.2–4.3**	3.61	5.99	4.57
*Brachyteles*	122	1.74	4.77	7.95	5.51
*Macaca*	89	2.1*	0.44	0.72	0.58
*Hylobates*	88–105	1.9–2.7*	No found	No found	No found
*Gorilla*	430–570	1.5–1.8*	No found	No found	No found
*Pan*	330–430	2.2–2.5*	No found	No found	No found
*Homo sapiens*	1.317	7.4–7.8*	12–13***	17–16	14–13

Data from *Galago senegalensis senegalensis*:[35]; *Macaca*: [36]*; Homo*: [37, 38]; encephalization quotient

*[39]

**[21]

***[13].

## Discussion

### Comparative morphology

The morphological data were compared with the anatomical descriptions of the brains of *Galago senegalensis senegalensis* (Strepsirrhini) and *Callithrix jacchus* (Platyrrhini); both exhibit lower complexity of brain structure than the primate species investigated in the present study—*Alouatta seniculus* (howler monkey) and *Ateles geoffroyi* (spider monkey), which are New World primates; *Macaca fascicularis*, *Macaca mulatta* (rhesus monkey), and *Papio cynocephalus* (baboons), which are Old World primates; *Pan troglodytes* (chimpanzees), which use tools; and humans (*Homo sapiens*).

The central sulcus is absent in *Galago senegalensis senegalensis* [35] and in *C*. *penicillata* (Fig 1). In *C*. *jacchus* [40] and *Saimiri ustus* (Fig 2), this sulcus is little developed. In *Sapajus libidinosus*, the sulcus shows higher length and sinuosity, little inclination, and location at the longitudinal midpoint of the telencephalon [11]. In the genera *Macaca*, *Papio*, *Pan*, and *Homo*, the central sulcus does not communicate with the lateral sulcus and has two curvatures, which are more inclined in *Pan* and *Homo* [36, 41, 42].

The frontal lobe of *Galago*, *Callithrix*, and *Saimiri* is characterized by the absence of sulci (Figs 1 and 2). In *Sapajus*, there are vertical and superior longitudinal sulci [11], which are respectively similar to the superior and inferior precentral sulci of *Alouatta* [41] and *Brachyteles* (Fig 4). In *Ateles*, the precentral sulcus is long, and arched and has a horizontal branch; it terminates adjacent to the central sulcus [41]. In *Macaca*, although there is divergence in the nomenclature—superior and inferior precentral sulci [37, 41] or superior and inferior arches [43–45]—there is consensus in the description of the trajectory. The superior precentral sulcus is short and shallow, whereas the inferior precentral sulcus is arched and C-shaped, with the concavity directed cranially, and has a horizontal branch similar to that found in *Ateles* [36, 41–44]. In *Papio* and *Pan*, these sulci are similar to those observed in *Macaca* [41] with a difference in nomenclature [46] have called these as the superior and inferior frontal sulci. In *Homo sapiens*, the precentral sulcus is segmented and delimits the precentral gyrus, which is related to motricity [37, 42].

A longitudinal sulcus on the inferior surface of the frontal lobe is similar in the primate species studied. In *Sapajus*, it is named inferior longitudinal sulcus [11] in *Ateles*, *Alouatta*, *Pan*, and *Papio*, it is called sulcus rectus [41]; in *Brachyteles*, it is known as inferior frontal sulcus; in *Macaca*, it is called frontal sulcus [45]. In *Homo sapiens*, superior and inferior frontal sulci are present, which increase the cortical surface area [37–42].

The postcentral sulcus in *Sapajus* [11] is delimited on the posterior portion of the postcentral gyrus and attached posteriorly to the lunatus sulcus, but in *Brachyteles* (Fig 4) and *Macaca*, this sulcus is short and does not join any other sulcus [36, 43, 45]. In *Alouatta*, *Ateles*, *Papio*, and *Pan*, it divides into superior and inferior postcentral sulci [41]. In *Homo sapiens*, it is parallel to the central sulcus and joins the intraparietal sulcus, similar to observations in *Pan* [37, 41, 42].

The convex aspect of the *Galago* and *Callithrix* telencephalon (Fig 1) is almost lissencephalic, except for the lateral sulcus, which begins at the height of the olfactory stria; delimited by the temporal lobe, it ascends in the posterior direction [35, 40]. In contrast, in *Saimiri*, the lateral sulcus (Fig 2) has a cranial curvature, and it terminates at the height of the longitudinal fissure. In *Sapajus* and *Brachyteles* (Fig 4), this sulcus terminates in the parietal lobe, such that there are one and two curvatures, respectively [11]. In *Alouatta* and *Ateles*, the lateral sulcus is continuous with the intraparietal sulcus; in the former, it ends in a bifurcation, whereas in the latter, it terminates close to the lunatus sulcus [41]. In *Macaca*, *Papio*, and *Pan*, the lateral sulcus courses to the parietal lobe with variations in the degree of sinuosity, which increases in these primate species, respectively [36, 41, 46, 47]. *Homo sapiens* shows the most highly developed lateral sulcus, which is very deep and has three branches: ascending, anterior, and posterior [42] report that the simplest configuration of the lateral sulcus in primates exposes the insular lobe, in contrast to the observations in humans, in which this sulcus is deeper and more developed [42].

The superior temporal sulcus is absent in *Galago* [35]. In *Callithrix* (Fig 1) and *Saimiri* (Fig 2), only the superior temporal sulcus is present on the temporal lobe. In *Sapajus*, *Ateles*, *Alouatta*, *Brachyteles* (Fig 4), *Papio*, *Pan*, and *Homo* sapiens, there are superior and inferior temporal sulci present. The superior temporal sulcus shows a more variable pattern across species. In *Sapajus*, this sulcus joins the lateral sulcus [11, 41]; in *Alouatta* and *Brachyteles* (Fig 4), it does not join other sulci. In *Macaca* and *Papio*, the superior temporal sulcus terminates in a bifurcation posterior and superior to the lateral sulcus [36, 41, 45]. Furthermore, in *Macaca*, there is a middle temporal sulcus, which is discontinuous and inferiorly delimits the superior temporal gyrus [45]. In *Pan*, the posterior part of the superior temporal sulcus joins the lunatus sulcus [46]. In *Homo sapiens*, the superior temporal sulcus runs parallel to the lateral sulcus and they rarely join [48]. The inferior temporal sulcus in *Pan* and *Homo sapiens* is discontinuous owing to several interruptions [37, 46].

The lunatus sulcus is absent in *Galago* and *Callithrix* (Fig 1), whereas in *Saimiri* (Fig 2), it is rudimentary, which makes it difficult to identify the parietal and occipital lobes on the convex aspect. In *Sapajus*, this sulcus is continuous with the postcentral sulcus [11]; in contrast, in *Alouatta*, the lunatus sulcus is located inferiorly to the lateral sulcus, does not characterize a specific lobe, and does not join any other sulcus [41]. In *Ateles*, it varies in shape (a straight path or arch) and joins the intraparietal sulcus [41]. In *Brachyteles* (Fig 4), the lunatus sulcus joins the lateral sulcus. In *Macaca*, it is continuous with the parieto-occipital sulcus and delimited by the angular gyrus in combination with the superior temporal sulcus [36, 45]. In *Papio* and *Pan*, the lunatus sulcus joins the intraparietal sulcus [41] and is located posteriorly, thus increasing the extension of the parietal lobe [49]. In *Homo sapiens*, this sulcus is absent in most cases; when present, it is segmented and displaced posteriorly and ventrally [34, 41, 46, 50]. According to Armstrong et al. [49], the difference in the position of the lunatus sulcus in primates may be associated with an increase in the associative cortex in the caudal part of the parietal lobe, whereas according to Holloway [50], this expansion contributed to the advancement of communication, use and creation of tools, social complexity, and long-term memory. In addition, it is observed that in humans, the primary visual cortex (Brodman’s area 17) and the peri-striatum (Brodman’s area 18) were pushed posteriorly, which displaced the lunatus sulcus to a more caudal position than that observed in *Pan*.

The organization of the parietal cortex is important as an area that shows large differences among primates, including humans. The parietal sulcus, as a hand–vision coordination area, may have its development intrinsically related to tool use behavior [51], as well as complex social behavior that may require a, possibly rudimentary, theory of mind [52]. The cortical area in the lateral sulcus also varies greatly among primates [53], with functions ranging from basic somatosensory to complex associative functions [54, 55], which may explain mirror self-recognition being observed in *Sapajus* and *Brachyteles*—an ability thought to be limited to humans and apes until recently—but not in other New World primates [56, 57].

In *Galago*, *Callithrix* (Fig 1), and *Saimiri* (Fig 2), the occipital lobe is lissencephalic; in contrast, *Sapajus* exhibits the inferior occipital sulcus (Fig 3). In *Alouatta*, *Ateles*, *Brachyteles* (Fig 4), *Macaca*, and *Papio*, there are superior and inferior occipital sulci. The inferior occipital sulcus is larger and deeper than the superior occipital sulcus and the latter is variable among species [41, 43, 45]. Moreover, in *Papio*, the lateral calcarine sulcus is present. In *Pan*, the inferior occipital sulcus is variable in its trajectory; it may join the middle temporal sulcus or occipitotemporal sulcus or not join any other sulcus [41]. In *Homo sapiens*, the path of the sulci and gyri on the convex surface of the occipital lobe varies more than that in the other lobes [48]. In general, there are two occipital sulci that subdivide the occipital lobe into three gyri: superior, middle, and inferior [42–48].

The medial region shows the presence of the cingulate sulcus. In *Galago*, it is short, whereas in *C*. *penicillata* (Fig 1) and *C*. *jacchus*, it is absent [33]. In *Saimiri* (Fig 2), its distal portion ascends slightly; however, in *Sapajus* (Fig 3), *Alouatta*, *Ateles*, *Brachyteles* (Fig 4), *Macaca*, *Papio*, and *Pan*, this sulcus ascends and forms the marginal branch [36, 38, 41, 43, 45, 47, 58]. In *Homo sapiens*, this sulcus is curved and has an approximate shape of a horizontal “s” [13]. In the posterior region, it divides into the paracentral and marginal branches, which are ascending, and the subparietal sulcus, which continues caudally [13, 42]. Paus [59] and Tamraz and Comair [34] have described anatomical variations in this sulcus in humans, such as duplication, interruptions in its trajectory, and formation of new branches in the telencephalon.

The rostral sulcus is absent in *Galago* [35], *Callithrix* (Fig 1) [40]; and *Saimiri* (Fig 2) and has a similar trajectory in *Sapajus* (Fig 3), *Brachyteles* (Fig 4), *Alouatta*, *Ateles*, *Macaca*, *Papio*, and *Pan* [36, 41, 44, 58]. In *Homo sapiens*, this sulcus separates the frontal gyrus medially into superior and inferior portions [13]. In some telencephalons, this sulcus may be joined to the cingulate sulcus, according to Paus [59] or appear duplicated, with the presence of an accessory rostral sulcus [34].

The sulcus of the corpus callosum is present in and has a similar trajectory in *Callithrix*, *Saimiri*, *Sapajus*, *Brachyteles* (Figs 1–4 respectively), and *Macaca*. However, in *Macaca*, the caudal portion of this sulcus joins the calcarine and hippocampus sulci [45]. In *Homo sapiens*, the sulcus of the corpus callosum and hippocampal sulcus are continuous, and they are separate from the calcarine sulcus [42, 48]. This sulcus has not been described for the other primate species considered in the present study.

In *Galago*, the calcarine sulcus consists of three small and continuous sulci: precalcarine, retrocalcarine, and paracalcarine. The first is near the hippocampal sulcus on the temporal lobe; the second is continuous with the cingulate sulcus; and the third goes to the occipital pole [35, 44]. In *Callithrix* (Fig 1), this sulcus goes to the occipital pole without dividing into the superior and inferior branches [40]. In *Saimiri*, *Sapajus*, and *Brachyteles* (Figs 2–4, respectively) *Macaca*, *Papio*, and *Pan*, the calcarine sulcus divides into the superior and inferior sulci on the occipital lobe [36, 41, 43]. Connolly [41] described the calcarine sulcus in *Alouatta* and *Ateles* as two sulci, the first is similar to that in *Sapajus*, and the second is the paracalcarine sulcus, which resembles a hook and originates in the occipital lobe before the parieto-occipital sulcus. *In Homo sapiens*, the calcarine sulcus bifurcates into the anterior and posterior calcarine sulci [34, 42].

In *Galago* and *Callithrix* (Fig 1), the parieto-occipital and subparietal sulci are absent [35, 47]. In *Saimiri* and *Sapajus* (Figs 2 and 3, respectively), the parieto-occipital sulcus does not join the calcarine sulcus, and the subparietal sulcus appears before the parieto-occipital sulcus or is continuous with it. In *Alouatta*, *Ateles*, and *Brachyteles* (Fig 4), the parieto-occipital sulcus is present but the subparietal sulcus is absent [38, 41]. Moreover, in *Ateles*, there is an accessory parieto-occipital sulcus [41]. In *Saimiri* and *Sapajus*, the subparietal sulcus is continuous with the lunatus sulcus on the convex surface of the cerebral hemisphere, and it is restricted on the parietal lobe; this criterion was used to differentiate it from the paracalcarine sulcus that is present in other primates [11]. In the genera *Macaca*, *Papio*, and *Pan*, the parieto-occipital and calcarine sulci do not join, and the subparietal sulcus is variable in its trajectory; in most cases, it is posteriorly connected to the parieto-occipital sulcus. Other possibilities are the absence of this sulcus or connections with other sulci [36, 41, 46]. In *Homo sapiens*, the parieto-occipital sulcus is located only on the medial aspect and terminates forming a right angle with the calcarine sulcus [42]. Although the parieto-occipital sulcus and calcarine sulcus appear continuous, they are separated by one or more small gyri [48]. The subparietal sulcus is derived from the cingulate sulcus and is not continuous with the parieto-occipital sulcus [42].

In *Galago*, the hippocampal sulcus courses in the posterior-to-anterior direction and contours the hippocampal tubercle on the temporal lobe [35]. In *Callithrix*, *Saimiri*, *Sapajus* (Figs 1–3 respectively), *Alouatta*, *Ateles*, *Brachyteles* (Fig 4), *Macaca*, *Papio*, and *Pan*, the hippocampal sulcus begins near the splenium of the corpus callosum and joins caudally to the sulcus of the corpus callosum and calcarine sulcus. Then, it continues to the temporal pole, where it terminates separating the para-hippocampal gyrus from the uncus [36, 38, 41, 46, 47]. In *Homo sapiens*, the trajectory of the hippocampal sulcus is similar to that in other primates, with the difference that the hippocampal sulcus is continuous only with the sulcus of the corpus callosum [42].

The collateral sulcus is absent in *Galago* and *Callithrix* (Fig 1) [35]. Connolly [41] reports that this sulcus has a very primitive organization in *Nycticebus* (family Lorisidae). In *Saimiri* (Fig 2), *Ateles*, and *Brachyteles* (Fig 4), the collateral and occipitotemporal sulci are continuous with each other, whereas in *Saimiri*, the collateral sulcus is connected to the calcarine sulcus and ends its trajectory close to the temporal pole. In *Sapajus* (Fig 3), *Alouatta*, *Macaca*, *Papio*, and *Pan*, this sulcus is well developed and exhibits two parts: the posterior part is joined to the calcarine sulcus is the collateral sulcus, and the anterior portion is joined to the occipitotemporal sulcus [36, 41, 43, 46, 47]. In *Homo sapiens*, the collateral and occipitotemporal sulci are well developed [34, 42, 48].

In *Galago*, *Callithrix*, and *Saimiri* (Figs 1 and 2), the rhinal sulcus is similar to shallow vascular depressions and separates the piriform lobe from the rest of the temporal lobe [35, 40, 44]. In *Sapajus* (Fig 3), *Brachyteles* (Fig 4), *Alouatta*, *Ateles*, *Macaca*, *Papio*, *Pan*, and *Homo sapiens*, this sulcus is evident and marks the boundary between the paleocortex and the neocortex in the temporal lobe [48]. In *Homo sapiens*, this sulcus is often continuous with the collateral sulcus, and this condition was also observed in four *Sapajus* brain specimens. It is suggested that this sulcus appeared in mammals after the ventral displacement of the pririform cortex due to the development of the neocortex [48].

### Allometric approach

The initial studies about the sulci and gyri of the cortex did not indicate any shared patterns among primates and humans [13]. After Gratiolet Pearce [60]^,^ the main sulci and gyri were defined for mammalian studies from the simplest to more complex (i.e., in humans).

The concepts of lissencephaly and gyrencephaly were refined and could be explained by the requirement of larger cortical areas (i.e., the circumvolutions of the brain) in relation to the volume of the cranial box (this approach is not universally accepted; for more detailed discussion, see [61]. Indeed, this relationship could be explained by a decrease of the encephalon by a power of 2/3 [61, 62]. That means, an object, when increased shows a non-linear relationship between area and volume; the volume increases by the third power, whereas the area increases by the second power [61, 62]. Accordingly, the energy expended for a biological structure to become larger and change its shape seems to be higher than the energy to increase its area without affecting the volume, according to previous evolutionary studies [61]. In this case, to save energy, an increase in the area of the brain, a soft tissue, is favored rather than an increase in bone mass or total volume of the cranium. Indeed, it seems easier to fold a soft brain tissue than to increase a cranial box with repercussions on the weight and animal movement and equilibrium, to say the least. However, this pattern is not observed in all mammals; in some mammalian species, the brain does not exhibit most gyri found in apes and humans.

Another aspect considered in the present study in relation to comparative brain analysis is EQ, an allometric measure associated with an increase in brain size, a subject of interest in many fields of neuroscience [49]. Indeed, brain size varies among animals, with larger and smaller animals having larger and smaller brains, respectively. The EQ is not a unanimously accepted concept [61]. There are discrepancies on the evolutionary scale in EQ values calculated according to the following formula that takes into consideration certain metabolic parameters [63]:

$$EQ=\frac{\mathrm{brain}\;\mathrm{weight}}{0.12\times {\left(\mathrm{body}\;\mathrm{weight}\right)}^{0.66}}$$

For instance, the EQ of dolphins (5.3) is larger than that of chimpanzee (2.2–2.5) [64] (Table 8). Accordingly, some authors do not consider EQ to be an ideal measure to estimate brain complexity [50, 61], but EQ seems to be precise for analysis of a close group, such as primates [64]. In general, apes and other primates show a higher EQ than other animals [60].

In fact, the differences in the brains are smaller for the phylogenetically closer primates and larger for the more phylogenetically distant ones [64, 65] however, EQ values calculated for prosimians using metabolic parameters are not different than those for monkeys [49] still presenting a statistically significant relationship between body weight and brain weight [66]

**Fig 5:**
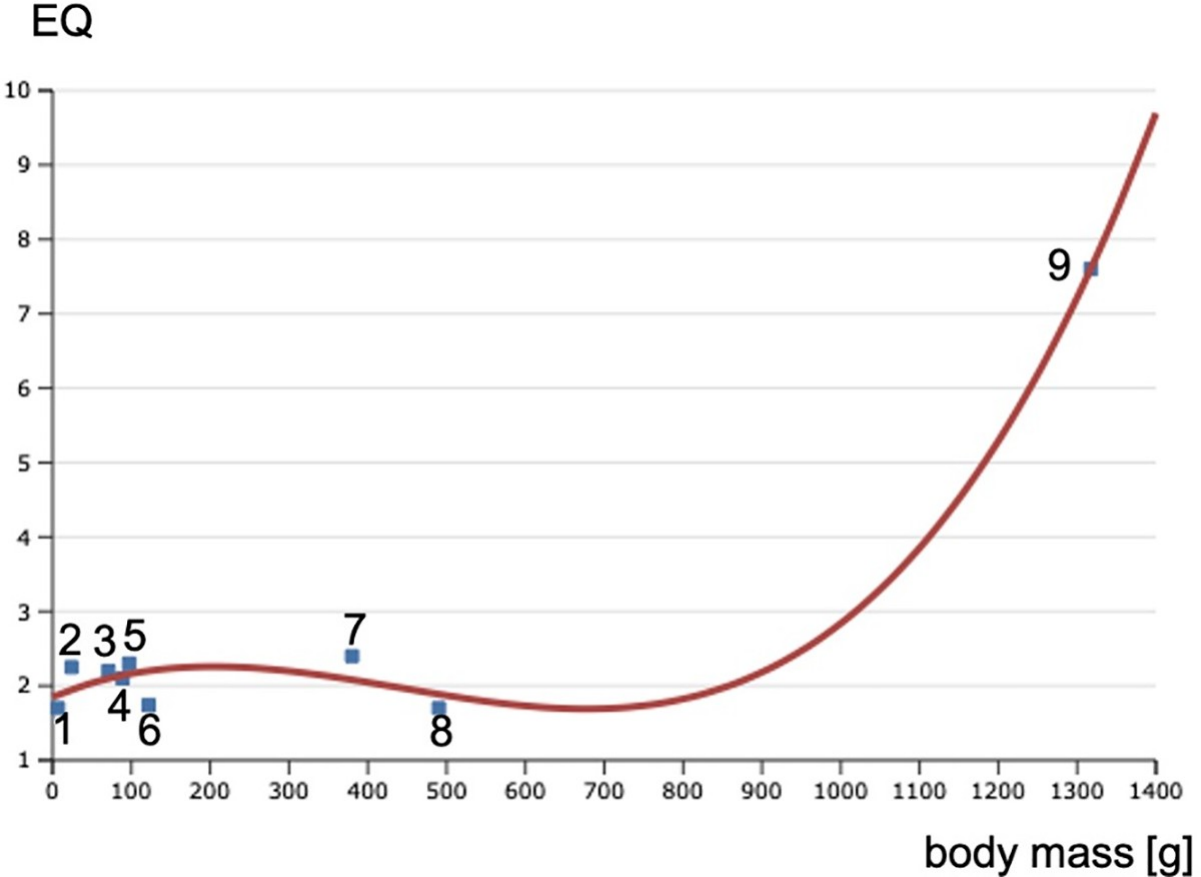
Polynomial regression of degree 3 shows a strong correlation between Encephalization Quotient (EQ) and brain mass (R^2^ = 0.98197). A linear regression was also performed (R^2^ = 0.081803). From left to right, the points correspond to *Callithrix* (1), *Macaca* (2), *Hylobates* (3), *Brachyteles* (4), *Saimiri* (5), *Sapajus* (6), *Pan* (7), *Gorilla* (8) *and Homo sapiens* (9).

In the present study, EQ values from some non-human primates to humans were plotted against cerebral mass; a complete linear regression of EQ was not observed, but a regression of degree 3 was observed. However, between *Callithrix* and *Gorilla*, a linear regression was observed (Fig 5).

For neotropical primates, there are discrepancies within the group, both for EQ and behavioral aspects, mainly for *Sapajus*. Indeed, many studies on *Sapajus* behavior suggest that they present high cognition comparable to that in chimpanzees [9, 14–28] and unexpectedly complex behavior for a New World primate, but a possible explanation may be provided on the basis of EQ (2.54 and 4.79 for *Sapajus* and *Cebus* [21]. These values of EQ for *Sapajus* have been discussed in detail by many authors on the basis of metabolism, suggesting that *Saimiri* and *Sapajus* present high EQ because of their high metabolic needs, whereas *Callithrix* and *Brachyteles* show lower metabolism [21]. In fact, the EQ values of the latter two are similar, and the EQ values of *Saimiri* and *Sapajus* are also similar (Table 8). Excluding the cited discrepancies owing to the metabolism rate, the EQ seems to increase proportionally with the phylogenetic scale but with discrepancies in the group of New World primates because the calculated EQ for *Saimiri* and *Sapajus* is larger than that for *Macaca*.

However, while the EQ of *Sapajus* may be justified by their high cognitive behavior, the same cannot be said for *Saimiri*. Considering the evolutionary scale of cognition, among the tropical primates, the EQ of *Saimiri* is inconsistent. In general, EQ seems to be an indicator for comparisons of cognition among primates, but the discrepancies must be considered. Calculating the EQ for other primates, mainly the less-studied New World primates [67] may indicate whether more or less differences would be found. The EQ for tropical primates is expected to be approximately 1.7, on the basis of the results obtained for *Callithrix* and *Brachyteles* (Table 8).

Another factor to evaluate are cerebral gyri and sulci. For humans and apes, the bilateral differences in some gyri and sulci indicate asymmetry of the cerebral hemispheres [13–42], an important evolutionary aspect associated with cognitive aspects of primates. In general, for some primates and other mammals, the asymmetry seems inconclusive [50].

Phylogenetically, the first sulcus to appear in the brain was the hippocampal sulcus, which delimits the archicortex, and the second was the rhinal sulcus, which separates the paleocortex from the neocortex [48]. Remarkably, these two sulci are also present in the almost lysencephalic primates such as *Galago* and *Callithrix* (Table 1) [35, 40] and persist in more complex brains such as those of apes [44] and humans. The highly conserved sulci in primate brains are the lateral, cingulate, calcarine, hippocampal, and rhinal sulci and longitudinal fissure [43] in *Callithrix*, the cingulate sulcus was absent (Table 1), but it is not appropriate to consider this primate as completely lissencephalic.

In general, differences in primate brains in comparison with other mammals are the increase in new parts of the encephalon, neocortex, and neencephalon and: 1) enhanced visual capabilities with concomitant complexity of the occipital cortex and optical pathways, generating a stereoscopic vision, 2) enhanced tactile sensibility in the limb extremities (hand/feet or pads), and 3) comparative diminution of olfactory capabilities [64].

Nevertheless, considering the difficulty in evaluating the cerebral differences with respect to the gross neuroanatomy, some measures could generate data to elucidate the differences among the primates’ brains, such as allometry.

In general, the permanent sulci and other variations present on both medial and lateral aspects in human brain may be observed because of the increased complexity due to the large cortical area; they are not so prominent in primates presenting a certain degree of gyrencephaly, such as the tropical primates investigated in the present study. Among the primates analyzed in this study, only the genera *Galago* [44] and *Callithrix* have no central sulcus. Indeed, their brains could be considered as lissencephalic to a certain extent, mainly on the convex aspect; however, on the medial aspect, at least the calcarine, rhinal, and hippocampal sulci are evident for all primates.

In gross anatomy, the degree of gyrencephaly is cited as a visual criterion of the convex aspect of the brain; however, it was purposed in this study. Moreover, a proportional measure between the straight and sinuous measurements for the main sulci for *Sapajus* [11] and for humans (Féré cited by [13] was used. The ratio of the straight by sinuous measurement, the degree of sinuosity, indicates the relative value for gyrencephaly for each sulcus and facilitates interspecies comparison; for sulci of the convex aspect, the ratio indicates more folding of the cortex. The absolute size of the sulci cannot be considered for interspecies analysis; however, the relative values permit a comparative analysis. The relative data [straight measurement/sinuous measurement] offers a direct and simple approach to estimate the relative cortical area to verify the hypothesis that the more gyrencephalic the brain is, the more gyrencephalic are the sulci, and to perform a comparative analysis with other genera.

**Fig 6:**
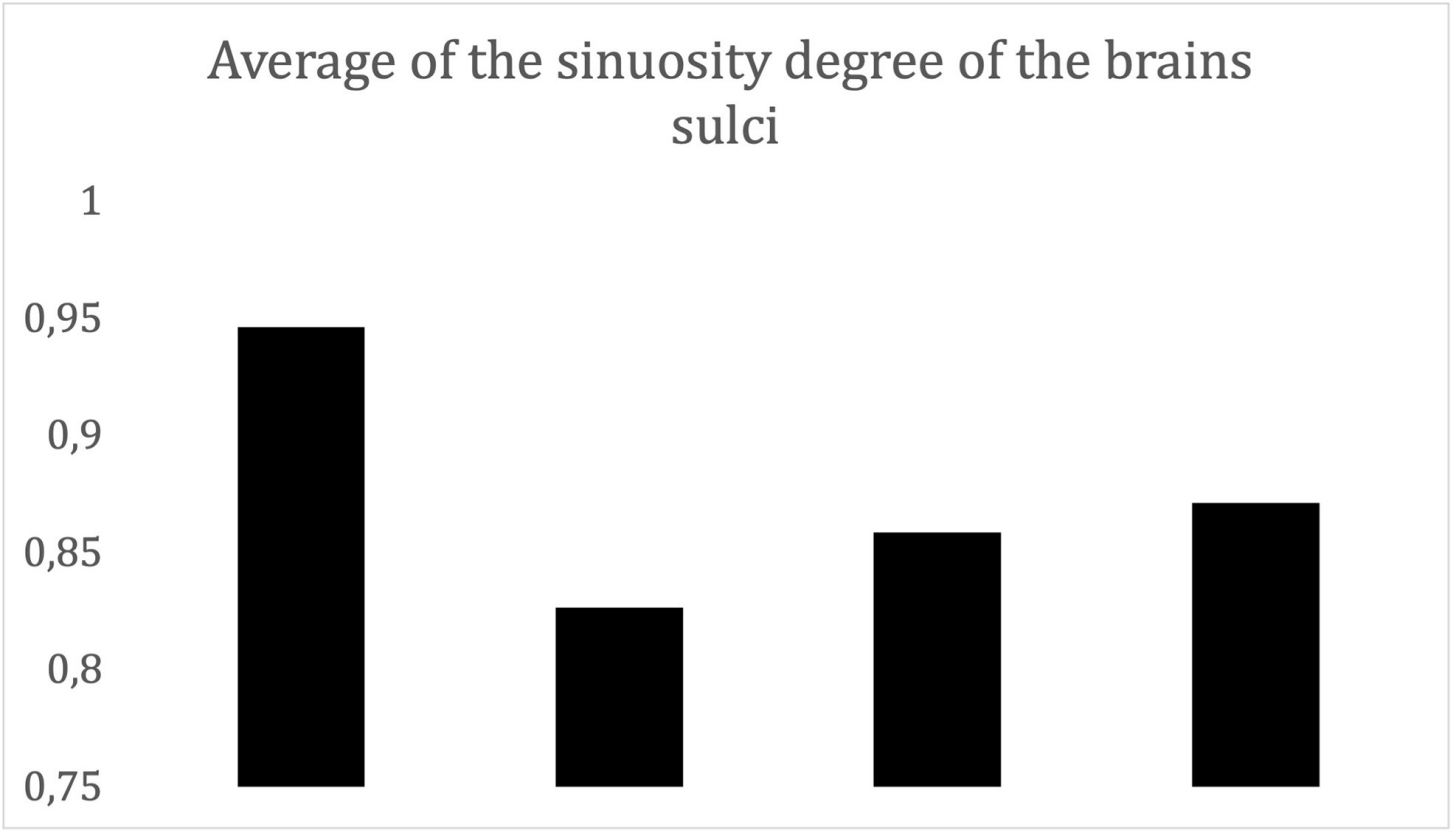
Mean degree of sinuosity of *Callithrix penicillata*, *Saimiri ustus*, *Sapajus libidinosus*, and *Brachyteles arachnoides*. The larger the value is, the smaller is the sinuosity.

According to our analysis, there was a high correlation between these data and visual observations for the level of gyrencephaly (Tables 1, 2, 6, and 7 and Fig 6). The degree of sinuosity, which represents the relative size of the sulci, shows lower values for more sinuous gyri. Accordingly, among the New World primates studied in this work (Table 6), the genus *Saimiri* exhibits a lower degree of gyrencephaly than *Sapajus* and *Brachyteles* on the basis of the sulci on the convex aspect, as can be observed in Figs 2–4 for *Saimiri*, *Sapajus* and *Brachyteles*, respectively. For humans, the highest degree of gyrencephaly was observed for the central sulcus (Table 6) as reported by Féré [13]. In *Callithrix*, the absence of most sulci indicates a high level of lissencephaly (Fig 1 and Tables 2, 3, and 6); in fact, on the convex aspect, only the lateral and temporal sulci were observed (Fig 1).

For the gyrencephaly analysis, we measured the brain structures and calculated the degree of sinuosity (Table 6) and also performed a mathematical analysis (Table 2), which is more objective and permits a more accurate evaluation of interspecies differences. For posterior studies, the degree of sinuosity of the sulci could be calculated for a better interspecies comparison of allometric aspects.

The distance from the superior and inferior edges of the central sulcus to both the occipital and frontal poles, taken separately, is an indirect indicator for the size of the anterior and posterior brain parts. These measurements were first performed by Féré, Passet [13] and Giacomini [68] for humans but have been scarcely used in neuroanatomical studies since. In the present study, the distance from the edges of the central sulcus to the cerebral poles was performed for dissected specimens of neotropical primates that presented a complete central sulcus. In this respect and other aspects evaluated in relation to gyrencephaly, *Sapajus* brain is closer to other New World primates.

## Material and methods

### Ethics statement

This work was approved by the Institutional Ethical Committee of the Federal University of Goiás (CoEP-UFG 81/2008, authorization from the IBAMA number 15275 for the proceedings detailed here) and of the Federal University of Tocantins (CEUA-UFT 23101-003220/2013-85). The details of animal use and welfare were in accordance with the recommendations of the Weatherall report, “The use of non-human primates in research.”

### Samples

Seven, two, one, and one adult cadaveric specimens, respectively, of *Sapajus* sp., *C*. *penicillata*, *Saimiri ustus*, and *B*. *arachnoides* were used in this investigation (Table 9). No animal was killed or euthanized for the purposes of this study: five *Sapajus* sp. individuals suffered accidental deaths in their natural habitat, and these specimens were donated by the Brazilian Institute of Environment and Renewable Natural Resources (IBAMA) to the collection of the Laboratory of Anatomy of the Federal University of Tocantins-Palmas-Tocantins (Brazil). The remaining *Sapajus libidinosus* specimens and the *C*. *penicillata* and *Saimiri ustus* specimens belonged to the Primates Center of University of Brasília and were donated to the University of Tocantins for research. The *B*. *arachnoides* specimen was studied at the Primatology Center of Rio de Janeiro (Guapimirim, RJ, Brazil). All specimens, except *B*. *arachnoides*, presently belong to the Laboratory of Anatomy of the Federal University of Tocantins, and access to them requires permission from its chairperson.

**Table 9:** General data of study specimens.

Species	Approximated age	Gender	Animal identification	Register number	Weight (g)	n	Origin
*Callithrix penicillata*	Adult	Female	CP 1	985141000603011	410.58	2	Primatology Center of UnB
		Male	CP 2	985141000602928	451.35		
*Saimiri ustus*	Elderly	Male	SU 1	F1	1070	1	Primatology Center of UnB
*Sapajus libidinosus*	Adult	Female	SP 1	985141000961333	2400	2	Primatology Center of UnB
		Male	SP 2	963007000018551	3200		
*Sapajus libidinosus*	Adult	Male	SP 3, SP4, SP 5, SP6, SP7	-	1000 a 3000	5	IBAMA-GO
*Brachyteles arachnoides*	Adult	Male	BA 1 (animal from a museum)	Animal from Museum CP 2506	7000	1	Primatology Center of Rio de Janeiro
Total number of specimens						11	

### Preparation of the animals for dissection

All procedures involving the animals were performed in accordance with the guidelines of the Brazilian Society of Animal Experimentation (COBEA). After trichotomy with a scalpel blade, the specimens were incubated in water at room temperature for 10–12 h. All specimens were perfused and fixed, by injecting 10% formaldehyde and 5% glycerin through the femoral vein. The animals were conserved in 10% formaldehyde in covered opaque containers to avoid penetration of light and evaporation of the preservative.

### Dissection and documentation

Brains were removed from the skull, weighed, and measured with a caliper and documented with a digital camera (Cannon, model EOS Kiss X3 lens 18–55). The nomenclature of gyri, sulci, and other structures was based on descriptions from previous studies on humans and other primates, mainly capuchins, which are New World primates [11]. Only the visible and reliably delimited structures were studied for each genus. The measurement of the major gyri in a straight line using a caliper and along the sinuosity using an inextensible string was performed to assess the degree of curvature, and the results were compared with the data from humans.

The degree of sinuosity was calculated using the following equation:

$$\mathrm{sinuosity}\;\mathrm{degree}=\frac{\mathrm{straight}\;\mathrm{measure}}{\mathrm{sinuous}\;\mathrm{measure}}$$

The major encephalon measure was performed longitudinally and transversely. The degree of gyrencephaly was assessed for the lobes and major sulci. The encephalization quotient (EQ) was calculated using the following equation:

$$EQ=\frac{\mathrm{brain}\;\mathrm{weight}}{0.12\times {\left(\mathrm{body}\;\mathrm{weight}\right)}^{0.66}}$$

**Fig 7:**
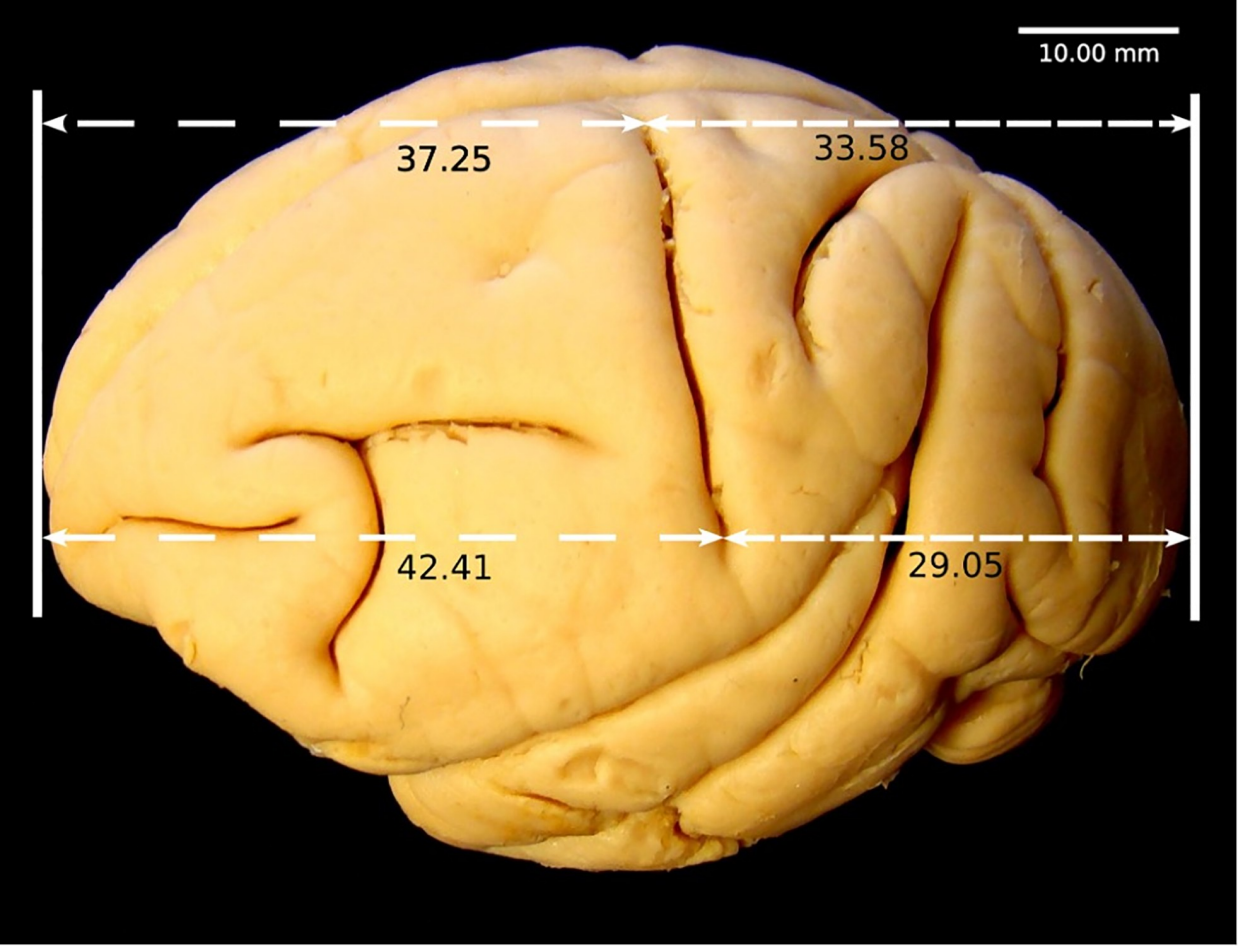
Photograph of the left convex aspect of the *Sapajus libidinosus* brain indicating the distance from the superior and inferior extremities of the central sulcus to the frontal and occipital poles. These measurements indicate the degree of inclination of the central sulcus.

The distance from the superior and inferior extremities of the central sulcus to the frontal and occipital poles was measured, when possible, to indicate the degree of inclination (ID) of this sulcus, and as an indirect measure of the frontal lobe (Fig 7), according to the following relation [13].

For brains of *C*. *penicillata* (Fig 1), *Saimiri ustus* (Fig 2), *Sapajus libidinosus* (Fig 3), and *B*. *arachnoides* (Fig 4), straight (Tables 4 and 5) and sinuos measurements of the sulci were performed (Tables 5–7). The ratio of the straight and sinuous measurements indicates a value that facilitates comparison of the sulcus size among different species and the degree of sinuosity of those sulci.

Statistical analysis of the frequency of the presence of assessed structures and central tendency measures (mean, standard deviation, and mean comparison) for the brain measurements was performed, and linear regression was performed for comparing the data of the studied species.

## Conclusions

In this study, we investigated the association between the gross anatomy with some behavior and physiology. The gross anatomy of the brain of *Sapajus* improved the data available for this primate; moreover, some concepts of allometry were used to analyze its complex behavior in an evolutionary context and the polynomial regression between brain size and body mass.

The genus *Sapajus* includes primates with a degree of gyrencephaly lower than that of Old-World primates but similar to that of New World primates, and this feature does not seem to account for the complex behavior in this genus, which often comparable to that of chimpanzees. Remarkably, despite this, the EQ of *Sapajus* is close to that of chimpanzees.

Research taking a histological approach may explain the unexpectedly high cognitive abilities observed in *Sapajus*. As the present study offers a limited analysis only on the basis of gross anatomy, certain measures, except for EQ, appear insufficient to explain the complexity of behavior in *Sapajus*. However, the evolutionary relationships deduced from gyrencephaly and EQ seem consistent, follow the already generally accepted notions.
